# Genetic architecture of thermotolerance traits in beef cattle: a novel integration of SNP and breed-of-origin effects

**DOI:** 10.3389/fgene.2025.1576966

**Published:** 2025-04-30

**Authors:** Gabriel A. Zayas, Camila Santos Rojas, Eduardo E. Rodriguez, Aakilah S. Hernandez, Ashley M. Beard, Fahad Rafiq, Kaitlyn M. Sarlo Davila, Raluca G. Mateescu

**Affiliations:** ^1^ Department of Animal Sciences, University of Florida, Gainesville, FL, United States; ^2^ Department of Animal Science, North Carolina State University, Raleigh, NC, United States; ^3^ Virus and Prion Research Unit, National Animal Disease Center, Agricultural Research Service, United States Department of Agriculture, Ames, IA, United States

**Keywords:** thermotolerance, PRLR, heat stress, GWAS, crossbreeding

## Abstract

**Background:**

Rising temperatures increasingly expose beef cattle to heat stress, reducing productivity and welfare, especially in tropical climates. Crossbreeding *Bos t.* t*aurus* and *Bos t.* i*ndicus* has emerged as a critical strategy to balance the production efficiency of taurine breeds with the superior thermotolerance of indicine breeds. Understanding the genetic architecture of thermotolerance traits is essential for improving heat resilience in beef cattle populations.

**Methods:**

Phenotypes for short hair length (SHL, undercoat) and long hair length (LHL, topcoat), sweat gland area (SGA), and thermal stress slope (TSS), a measure of body temperature fluctuations under heat stress, were collected from 3,962 crossbred Angus-Brahman heifers. Heifers were genotyped, and breed-of-origin (BOA) for each marker was determined using LAMP-LD. Genome-wide association studies were conducted using SNP-only, BOA-only, and integrated SNP + BOA models to identify quantitative trait loci (QTLs) associated with thermotolerance traits. Genes in QTL regions were used for functional enrichment analysis using Gene Ontology (GO) and KEGG pathways.

**Results:**

Significant QTLs for SHL and LHL were identified on BTA20, overlapping the *PRLR* gene. A QTL on BTA19 for SHL and LHL was driven solely by BOA effects, with Brahman BOA associated with shorter hair lengths. For SGA, six suggestive QTLs were detected, predominantly linked to Angus-derived alleles associated with reduced sweat gland area. For TSS, a significant QTL on BTA1 exhibited a strong BOA effect, with Angus BOA associated with higher TSS values, indicative of reduced thermoregulatory efficiency. Integrated SNP + BOA models provided greater resolution and revealed novel QTLs compared to single-effect models. Functional enrichment using GO and KEGG identified MAPK and estrogen signaling pathways in both LHL and TSS, indicating potential overlap in the biological processes influencing hair length and thermoregulation.

**Conclusion:**

This study demonstrates the value of integrating BOA with SNP-based models to uncover the genetic architecture of thermotolerance traits in beef cattle. By better capturing breed-specific contributions, these findings enhance our understanding of thermoregulation and provide actionable insights for improving heat resilience in cattle.

## 1 Background

Rising global temperatures have introduced challenges for beef production, as approximately 80% of cattle globally experience heat stress conditions for at least 30 days per year, with the severity being greater in tropical regions (Carvajal et al., 2021). Heat stress adversely affects productivity, health, and welfare in cattle, necessitating strategies to enhance thermotolerance. Thermotolerance refers to the cattle’s ability to regulate body temperatures whilst maintaining production in the presence of heat stress conditions. One promising approach to improving thermotolerance in cattle is crossbreeding *Bos t. taurus* and *Bos t. indicus* breeds. *Bos t. taurus* breeds are valued for their production efficiency, while *Bos t. indicus* breeds are generally very heat tolerant. By combining these attributes, crossbreeding offers a practical strategy to improve both productivity and thermotolerance in beef cattle. The evolutionary history of *Bos t. indicus* and *Bos t. taurus* cattle are quite different, as each originated from distinct domestication events and experienced different selection pressures and adaptations (Bolormaa et al., 2013; Koufariotis et al., 2018). *Bos t. taurus* breeds have been selectively bred for a variety of traits that optimize production, leading to the development of numerous specialized breeds, each selected for specific purposes, such as meat quality, growth rate, or milk production. In contrast, *Bos t. indicus* cattle have undergone selection for resilience in tropical climates, with distinct physical adaptations for regulating body temperature in high heat and humidity (Utsunomiya et al., 2019; Verdugo et al., 2019; Mateescu et al., 2020). These adaptations include a larger skin surface area, longer ears (Utsunomiya et al., 2019), slicker hair coats (Sarlo Davila et al., 2019), and larger sweat glands for efficient heat dissipation (Mateescu et al., 2023; Hernandez et al., 2024). The observable morphological differences between *Bos t. taurus* and *Bos t. indicus* cattle are a direct reflection of underlying variations in their genetic architecture (Koufariotis et al., 2018). Crossing breeds from these two different origins can result in cattle that truly embody the “best of both worlds.”

Understanding the genetic basis of thermotolerance in beef cattle is essential for improving performance in hot and humid environments. The mixing of *Bos t. taurus* and *Bos t. indicus* genetic backgrounds often results in greater variation in thermotolerance traits, creating opportunities to identify and select individuals with superior heat tolerance. Previous studies have described the heritability of hair length traits (Sarlo Davila et al., 2019), sweat gland area (Mateescu et al., 2023; Hernandez et al., 2024) and core body temperature traits (Sarlo Davila et al., 2019) in crossbred Angus-Brahman cattle, reporting high to moderate heritabilities. This variability enhances the potential for identifying genetic regions influencing thermotolerance, as greater genetic and phenotypic variation increases the power to detect associations between genetic markers and traits of interest. However, differences in the evolutionary histories of *Bos t. taurus* and *Bos t. indicus* breeds result in differences in linkage disequilibrium (LD) patterns, which present challenges for genomic analyses. In crossbred and composite populations, the LD structure is often less stable, as it represents a mixture of the parental breeds’ LD patterns. This can reduce the accuracy of identifying quantitative trait loci (QTL), as these analyses rely on LD between genetic markers and causal variants. Addressing these challenges requires advanced genomic approaches that take into account crossbred populations unique genetic structure.

Breed of Origin of Alleles (BOA) offers an innovative solution by capturing breed-specific genomic contributions (Vandenplas et al., 2016; Claudia et al., 2019). BOA assigns each genetic marker to its ancestral breed, enabling simultaneous modeling of SNP and BOA effects. This approach enhances the precision of genomic evaluations by accounting for the distinct genetic architectures of the contributing breeds, thereby facilitating more accurate predictions of traits (Guillenea et al., 2023). By accurately assigning each allele to its breed of origin, BOA enables researchers to discern how breed-specific genetic contributions influence various traits in crossbred animals. This joint approach ensures that QTL identification accounts for both genomic variation and breed-specific contributions to trait expression. Previous studies in crossbred Angus-Brahman cattle have identified BOA effects associated with economically relevant traits such as marbling and carcass weight (Zayas et al., 2024b). However, these studies modeled SNP and BOA effects separately, which can limit the ability to detect meaningful genomic associations. Fitting both SNP and BOA information simultaneously, can improve the robustness and accuracy of QTL detection in crossbred cattle.

This study aimed to identify QTLs associated with thermotolerance traits in beef cattle, including short hair length (SHL), long hair length (LHL), sweat gland area (SGA) and thermal stress slope (TSS). Genome-wide association studies (GWAS) were conducted modeling SNP and BOA effects simultaneously. This approach was compared to traditional SNP-only and BOA-only models to identify genomic regions essential for thermotolerance traits.

## 2 Methods

### 2.1 Animals and phenotypic measurements

The research protocol for this study was approved by the University of Florida Institutional Animal Care and Use Committee (IACUC #202200000019). The study population consisted of 3,962 commercial Brangus heifers and Angus-Brahman crossbred heifers from the University of Florida’s multibreed Angus-Brahman (MAB) herd. A total of 2,772 commercial Brangus heifers were sampled from the Seminole Tribe of Florida, Inc., located in Okeechobee, FL, over the years 2016–2019. Additionally, 669 commercial Brangus heifers were sourced from Williamson Cattle Company in Chiefland, FL, during 2021 and 2022. The UF MAB heifers comprised 521 individuals collected in 2017, 2018, 2023, and 2024.

Phenotypic measurements and DNA samples, collected via tissue or blood, were obtained during the summer months. Heifers were sampled in groups of 70–200, with a total of 22 groups. All heifers within a given group and year were from the same cohort and were of similar ages, reflecting uniform management practices. Sampling occurred during the summer months to capture phenotypic data relevant to heat stress under natural environmental conditions. Four traits were measured to assess thermotolerance traits: SHL, LHL, SGA and TSS.

#### 2.1.1 Short and long hair length

Hair samples were collected from the shoulder, 10 cm below the spine and halfway along the horizontal axis of each animal, as described in previous studies (Sarlo Davila et al., 2019; Davila et al., 2020). Samples were spread on white paper and photographed alongside a ruler, which was used to convert pixels to millimeters. Hair lengths were measured using ImageJ software (Schindelin et al., 2012). The undercoat (SHL) and topcoat (LHL) were assessed by averaging five short hairs and five long hairs from each animal. Outliers, defined as hairs with lengths exceeding three standard deviations from the mean, were excluded to ensure accurate representation of typical hair length.

#### 2.1.2 Sweat gland area

Skin biopsies were collected from the shoulder, 10 cm below the spine and midway along the horizontal axis of each animal, following previously described methods (Mateescu et al., 2023; Hernandez et al., 2024). Briefly, the skin was disinfected with 70% ethanol and a 2% chlorhexidine solution, then sprayed with 4% lidocaine topical anesthetic. A 6-mm biopsy punch (Miltex Inc., York, PA, USA) was used to obtain the sample, which was then fixed in 10% formalin for 16–24 h at room temperature. After fixation, biopsies were dehydrated in 70% ethanol, embedded in paraffin, and sectioned at a thickness of 7 μm. Sections were mounted on slides, stained with Harris-Eosin Hematoxylin, and digitized using a Nikon T3000 inverted phase microscope equipped with NIS Image Elements software. Images were captured at ×40 magnification and analyzed using ImageJ software (Schindelin et al., 2012). SGA was measured as the total area occupied by sweat glands within a 1,100 × 1,100-pixel image section. Measurements were converted from pixels to millimeters using a scale of 1,000 pixels = 2.145 mm.

#### 2.1.3 Thermal stress slope

Core body temperature was measured as vaginal temperature using iButton data loggers (type DS1922L, temperature range −40°C to 85°C, accuracy ±0.5°C, resolution ±0.0625°C; Maxim Integrated, San Jose, CA) during a 5-day period during the summer months across each year of collection. iButtons were attached to blank controlled internal drug-release (CIDR) devices and inserted into the vagina of each heifer. Before deployment, all iButtons were calibrated to ensure measurement accuracy and preprogrammed to record body temperature at 15-min intervals on a 24-h cycle. Data from the first and last day, corresponding to CIDR insertion and removal, were excluded.

Environmental conditions, including temperature, humidity, dew-point temperature, and black globe temperature, were continuously monitored using HOBO data loggers (Onset Computer Corp., Bourne, MA). Hourly averages of body temperature were matched with the corresponding hourly averages of THI recorded during the same time. The temperature-humidity index (THI) was calculated using the formula:

$$THI=\left(1.8\times TT+32\right)\;–\;\left[0.55\;–\;0.0055\times RH)\times \left(1.8\times T-26\right)\right]$$

where T is air temperature (°C) and RH is relative humidity (%). This equation has been validated as an indicator of heat stress in livestock (Animals, 1971; Dikmen and Hansen, 2009).

To quantify the impact of daily thermal fluctuations on beef cattle under heat stress conditions a novel metric was used. TSS was calculated for each individual as the ratio of the diurnal variation in body temperature—determined by the difference between maximum and minimum body temperatures within a 24-h period—to the THI load for the same day. The THI load is defined as the cumulative deviation of the THI above a baseline threshold of 70, reflecting the intensity and duration of thermal stress experienced by the animals. To ensure consistency in assessing heat stress, a single day was selected for each group of animals. These days were chosen based on high THI load and the absence of rain events, which could otherwise confound the effects of thermal stress. The following formula was used to calculate TSS:

$$TSS=\frac{VT_{\max}-{VT}_{\min}}{\sqrt{\sum_{i=1}^{n}\left({THI}_{i}-70\right)}}\times 100$$

where VT_max_ and VT_min_ represent the maximum and minimum vaginal temperatures recorded during the day, and THI_i_ represents the hourly THI. This approach quantifies an individual’s thermoregulatory capacity under naturally occurring environmental heat stress, offering a precise metric for genetic and phenotypic analysis.

### 2.2 Genotyping and quality control

Genomic DNA was extracted from either tissue or blood samples using the QIAamp DNA Mini DNA kit (Qiagen, Valencia, CA, United States) following the manufacturer’s protocol and stored at −20°C. Genotyping was conducted using the Bovine GGP F250 array (GeneSeek, Inc., Lincoln, NE) containing 221,115 SNPs enriched with functional variants including non-synonymous, frameshift, and stop mutations. Only autosomal SNP were mapped to the ARS-UCD1.2 assembly and retained for further analysis. Quality control (QC) filtering was performed with PLINK2 (Chang et al., 2015). QC filters excluded animals with a genotype completion rate below 90%, markers with a minor allele frequency below 5% and a genotype call rate below 99%.

### 2.3 Breed of origin assignment

Local Ancestry in adMixed Populations (LAMP-LD) was used to infer percentages of local ancestry of each animal (Paşaniuc et al., 2009; Baran et al., 2012). LAMP-LD uses hidden Markov models of haplotype diversity of the ancestral/purebred populations within a window-based framework to trace the origin of alleles in the admixed population (Paşaniuc et al., 2009; Baran et al., 2012). Purebred Angus and Brahman cattle from the University of Florida’s MAB herd were used to represent the purebred populations for the LAMP-LD Analysis. A total of 123 purebred Angus cattle and 406 purebred Brahman cattle were used as the reference population. Only markers with an allele frequency difference (AFD) ≥5% between purebred population were used, to ensure sufficient differentiation between breeds. After QC filtering and AFD filtering a total of 107,153 markers remained for further analysis. The local ancestry results from LAMP-LD were subsequently used to infer the BOA. The BOA of the resulting 107,153 SNPs were then converted into a pseudo-genotype format using in-house scripts, where 0 represented homozygous Angus (AA), 1 represented the heterozygote state (AB/BA) and 2 represented homozygous Brahman (BB). The pipeline utilized is available under https://github.com/gzayasPR/BOA_Estimation (Zayas et al., 2024b), along with further details on data processing and management (Tange, 2011; Knaus and Grünwald, 2017; R Core Team, 2023; Barrett et al., 2024; Wickham et al., 2024).

### 2.4 Genome-wide association studies

GWAS was conducted to assess SNP and BOA effects on the phenotypic trait of interest using the following linear mixed models:

y=Xb+SNP+Zg+e

y=Xb+BOA+Zg+e

y=Xb+SNP+BOA+Zg+e

where: *y* denotes a vector of phenotypic records, SNP represents the SNP effect, modeled as a fixed effect encoded as a linear covariable as 0, 1, 2 (0 = homozygous for the major allele, 1 = heterozygous and 2 = homozygous for the minor allele). BOA is the Breed of Origin of Allele effect, as a fixed effect encoded as a linear covariable as 0, 1, 2 (0 = homozygous for the Angus BOA, 1 = heterozygous BOA and 2 = homozygous for the Brahman BOA). **X** is the incidence matrix, connecting phenotypic records to fixed effects, *b* is a vector of the fixed effects, including a categorical collection group, **Z** is the incidence matrix linking random genetic effects to phenotypes, g is a vector of random animal additive genetic effects, distributed as $g\;∼\;N\left(0,G\sigma_{g}^{2}\right)$ , where $\sigma_{g}^{2}$ is the additive genetic variance, *e* is a random residual vector, distributed $e\;∼\;N\left(0,I\sigma_{e}^{2}\right)$ , with $\sigma_{e}^{2}$ signifying residual variance **I** the identity matrix.

The genomic relationship matrix G was constructed based on the method proposed by VanRaden (VanRaden, 2008)

$$G=\frac{ZZ^{\prime }}{2\sum p_{i}\left(1-p_{i}\right)}$$

To minimize bias from proximal markers, a Leave-One-Chromosome-Out (LOCO) approach was implemented, where the G matrix for each SNP was constructed using markers from all chromosomes except the one harboring the tested SNP

The single-marker GWAS was performed using the lmm.diago function from the gaston package (Perdry and Dandine-Roulland, 2023) in Rv4.4 (R Core Team, 2023). The lmm.diago function in the gaston package was chosen for its computational efficiency in handling large genomic datasets and its robust implementation of linear mixed models for GWAS. Marker significance was determined via single or joint Wald tests, and results were represented as -log(p-values).

To correct for multiple testing, a Bonferroni correction was implemented with an alpha level of 0.1 to control for family-wise error rate. In addition, a suggestive threshold was set at 1/number of tests to identify markers that showed potential associations but did not meet the stringent genome-wide significance criteria. GWAS results were processed using custom R scripts (R Core Team, 2023) and plotted using ggplot2 (Wickham, 2009). For mapping SNPs to specific genes, the Ensembl version 107 (Cunningham et al., 2022) and the ARS-UCD 1.2.0 genome assembly (Zimin et al., 2009) were utilized. Pipeline for GWAS can be found in https://github.com/gzayasPR/BOAxSNP-GWAS.git.

### 2.5 Functional enrichment analysis

To investigate the biological relevance of genomic regions associated with thermotolerance traits, functional enrichment analyses were performed using gene sets located near significant and suggestive SNPs. For each trait, genomic regions ±50 kb around the lead SNPs were annotated using Ensembl’s *Bos taurus* gene build (release July 2023) via the biomaRt package in R (Durinck et al., 2005; Durinck et al., 2009; R Core Team, 2023).

Gene Ontology (GO) and Kyoto Encyclopedia of Genes and Genomes (KEGG) pathway enrichment analyses were performed using the clusterProfiler package in R, with *Bos taurus* as the reference organism (Yu et al., 2012; Xu et al., 2024; Yu, 2024). GO enrichment was conducted across all three categories: Biological Process, Molecular Function, and Cellular Component. KEGG enrichment was carried out to identify overrepresented signaling and metabolic pathways. In both analyses, terms were considered significant at a false discovery rate (FDR) threshold of <0.1, and only gene sets with a minimum of three genes were included. All analyses were implemented in R (R Core Team, 2023), and trait-specific GO and KEGG results were saved as CSV files for reproducibility.

## 3 Results and discussion

The findings of this study highlight the value of BOA in understanding the genetic architecture of thermotolerance traits, in crossbred beef cattle. Unlike single-breed systems, crossbred cattle are characterized by unique patterns of LD that arise from the combination of parental breeds with distinct evolutionary histories. These complex LD patterns often present challenges for traditional SNP-based methods in detecting QTLs. BOA overcomes these limitations by leveraging ancestral information, enabling more precise identification of genomic regions and their associated effects.

### 3.1 Phenotypes

Table 1 provides descriptive statistics for all traits analyzed. The traits exhibited considerable variability, as indicated by their coefficient of variation, which ranged from 28.24% (LHL) to 43.57% (SGA). The wide range of values for each trait underscores the biological variation within the dataset, highlighting its potential to provide meaningful insights into association analyses and potential selection.

**TABLE 1:** Summary of phenotypic data.

Trait	N	Mean	StDev	Min	Max	CV, %
SGA, μm^2^	2,562	299.56	130.53	6.41	1153.77	43.57
TSS	3,962	7.83	2.45	0.57	21.72	31.28
LHL, mm	3,513	15.38	4.34	3.84	38.04	28.24
SHL, mm	3,588	7.06	2.49	0.18	23.11	35.31

Summary of the phenotypic data for sweat gland area (SGA), thermal stress slope (TSS), long hair length (LHL), and short hair length (SHL). Data includes the number of animals (N), mean values, standard deviation (StDev), minimum and maximum values, and the coefficient of variation (CV, %)

### 3.2 GWAS long and short hair length

**FIGURE 1:**
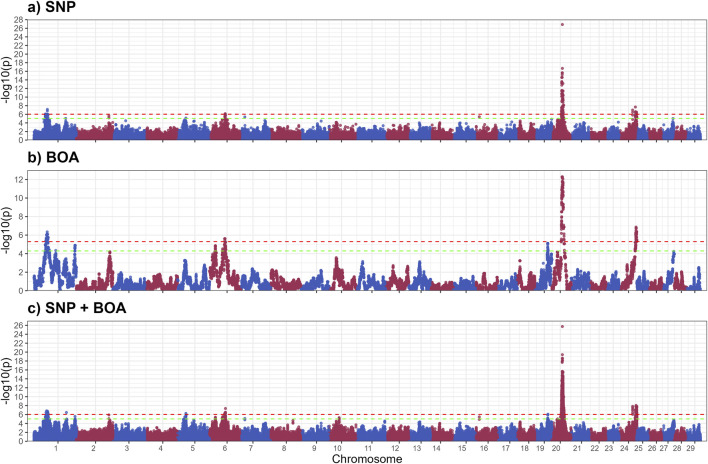
Manhattan plots from GWAS for long hair length using **(a)** SNP-based, **(b)** BOA-based, and **(c)** SNP + BOA combined analyses. The -log10(p-values) are plotted across chromosomes, with green and red dashed lines representing the suggestive threshold (1/number of SNPs) and genome-wide significance threshold (Bonferroni correction, α = 0.1), respectively.

Figure 1 illustrates the GWAS Manhattan plot for the LHL trait, with the corresponding QTL intervals and their significance levels summarized in Table 2. For detailed information of SNP and BOA effects see Supplementary Table S1. In total, thirteen QTL were identified for LHL across various chromosomes (BTA). Of these, eight QTL, located on BTA 1 (42.7–57.2 Mb and 118.6–154.0 Mb), BTA 5, BTA 6 (55.7–60.8 Mb), BTA 19, BTA 20, and BTA 24, exceeded the Bonferroni significance threshold. Additional QTL on BTA 2, 6 (21.7–22.5 Mb), 7, 10, and 16 reached suggestive levels of significance.

**TABLE 2:** Summary of significant and suggestive QTLs for traits related to short hair length, long hair length, sweat gland area, and thermal stress slope.

Trait	BTA	Start (bp)	End (bp)	Model	Effect	Candidate genes	Significance
**TSS**	**1**	**35,693,271**	**37,453,604**	**2,3**	**BOA**	***CSNKA2IP, EPHA3***	**Significant**
**TSS**	**1**	**40,669,335**	**42,196,332**	**2,3**	**BOA**	***EPHA6,ARL6, CRYBG3,RIOX2***	**Significant**
**TSS**	**1**	**44,026,431**	**58,543,037**	**1,2,3**	**BOA,SNP**	***FILIP1L,ALCAM, DCBLD2,NFKBIZ***	**Significant**
**TSS**	**1**	**72,045,065**	**78,420,586**	**2,3**	**BOA**	***PPP1R2,ATP13A3, GP5,CPN2***	**Significant**
TSS	2	126,787,596	127,293,109	1,2,3	SNP,BOA	*CRYBG2,MAN1C1*	Suggestive
TSS	10	36,260,837	36,260,837	3	BOA	*RAD51*	Suggestive
TSS	18	18,826,600	18,826,600	3	Joint	*TENT4B*	Suggestive
TSS	28	30,938,556	32,325,901	2,3	BOA	*SAMD8,VDAC2, COMTD1,LRMDA*	Suggestive
TSS	28	41,542,249	41,542,249	1	SNP	*BMPR1A*	Suggestive
**SGA**	**5**	**46,743,902**	**49,018,522**	**2,3**	**BOA**	***GRIP1,HELB, IRAK3,MSRB3, LEMD3,WIF1***	**Significant**
SGA	5	85,247,656	85,328,110	3	BOA	*BCAT1*	Suggestive
SGA	7	4,058,548	4,058,548	1	SNP	*NCAN,NR2C2AP, RFXANK, BORCS8*	Suggestive
**SGA**	**12**	**84,263,574**	**86,131,903**	**1,2,3**	**SNP,BOA**	***COL4A1,NAXD, ARHGEF7***	**Significant**
SGA	15	31,245,080	31,274,286	1,3	SNP	*GRIK4,ARHGEF12*	Suggestive
SGA	15	61,734,699	61,734,699	1,3	SNP	*DCDC1*	Suggestive
SGA	19	19,845,442	19,845,442	1	SNP	*POLDIP2,TMEM199,SEBOX*	Suggestive
**SGA**	**20**	**33,037,580**	**33,760,010**	**2,3**	**BOA**	***PTGER4***	**Significant**
SGA	21	68,139,315	68,139,315	3	SNP	*MARK3*	Suggestive
SGA	26	33,892,534	33,892,534	1	SNP	*TCF7L2,HABP2, NRAP*	Suggestive
**SHL**	**3**	**19,750,095**	**22,382,100**	**1,2,3**	**SNP,BOA**	***ECM1***	**Significant**
SHL	3	26,066,448	26,161,437	2,3	BOA	*VTCN1,TTF2*	Suggestive
**SHL**	**12**	**75,287,080**	**75,287,080**	**1,3**	**SNP**	***IPO5,FARP1,STK24***	**Significant**
SHL	18	56,713,940	61,281,386	2.3	BOA	*SYT3*	Suggestive
SHL	19	59,872,022	63,432,318	2,3	BOA	*ABCA5,PRKAR1A, WIPI1,PRKCA*	Suggestive
**SHL**	**20**	**36,018,194**	**44,480,511**	**1,2,3**	**SNP**	***PRLR***	**Significant**
SHL	24	58,163,445	58,163,445	3	BOA	*SEC11C*	
**LHL**	**1**	**42,735,608**	**57,217,658**	**1,2,3**	**SNP,BOA**	***FILIP1L,ALCAM, CBLB***	**Significant**
**LHL**	**1**	**118,603,687**	**153,989,230**	**2,3**	**BOA**	***BTD***	**Significant**
LHL	2	121,298,338	121,318,740	1,3	SNP	*DCDC2B,CCDC28B*	Suggestive
**LHL**	**5**	**27,632,416**	**32,334,842**	**3**	**Joint**	***OR and KRT gene clusters***	**Significant**
LHL	6	21,722,915	22,460,239	2,3	BOA	*CENPE, NFKB1*	Suggestive
**LHL**	**6**	**55,691,468**	**60,780,598**	**1,2,3**	**SNP,BOA**	***RELL1,WDR19***	**Significant**
LHL	7	13,923,322	13,923,322	1,2	SNP	*OR7H5P,OR7E201,*	Suggestive
LHL	10	37,495,328	37,498,039	3	BOA	*PLA2G4D*	Suggestive
LHL	16	12,426,704	12,426,704	1,3	SNP	*RO60,UCHL5*	Suggestive
**LHL**	**19**	**47,800,394**	**49,016,105**	**2,3**	**BOA**	***ACE,MAP3K*** *3* ***, CEP95***	**Significant**
**LHL**	**20**	**35,087,221**	**45,382,615**	**1,2,3**	**SNP,BOA**	***PRLR***	**Significant**
**LHL**	**24**	**46,439,947**	**46,441,868**	**1,3**	**SNP**	***ST8SIA5,PIAS2***	**Significant**
**LHL**	**24**	**55,570,233**	**62,261,715**	**1,2,3**	**SNP,BOA**	***FECH, NEDD4L, ALPK2,PHLPP***	**Significant**

Breed of Origin categories: BB (Brahman-Brahman), AB (Angus-Brahman), and AA (Angus-Angus). This table summarizes significant and suggestive QTLs identified for thermal stress slope (TSS), sweat gland area (SGA), short hair length (SHL), and long hair length (LHL). Columns include the trait, chromosome (BTA), genomic interval (QTL Start and End), GWAS model type (1: SNP effects, 2: BOA effects, 3: SNP and BOA effects), effect type (SNP, BOA, or Joint), candidate genes, and significance based on Bonferroni correction (significant) or suggestive thresholds (suggestive). Significant candidate genes are highlighted in bold.

**FIGURE 2:**
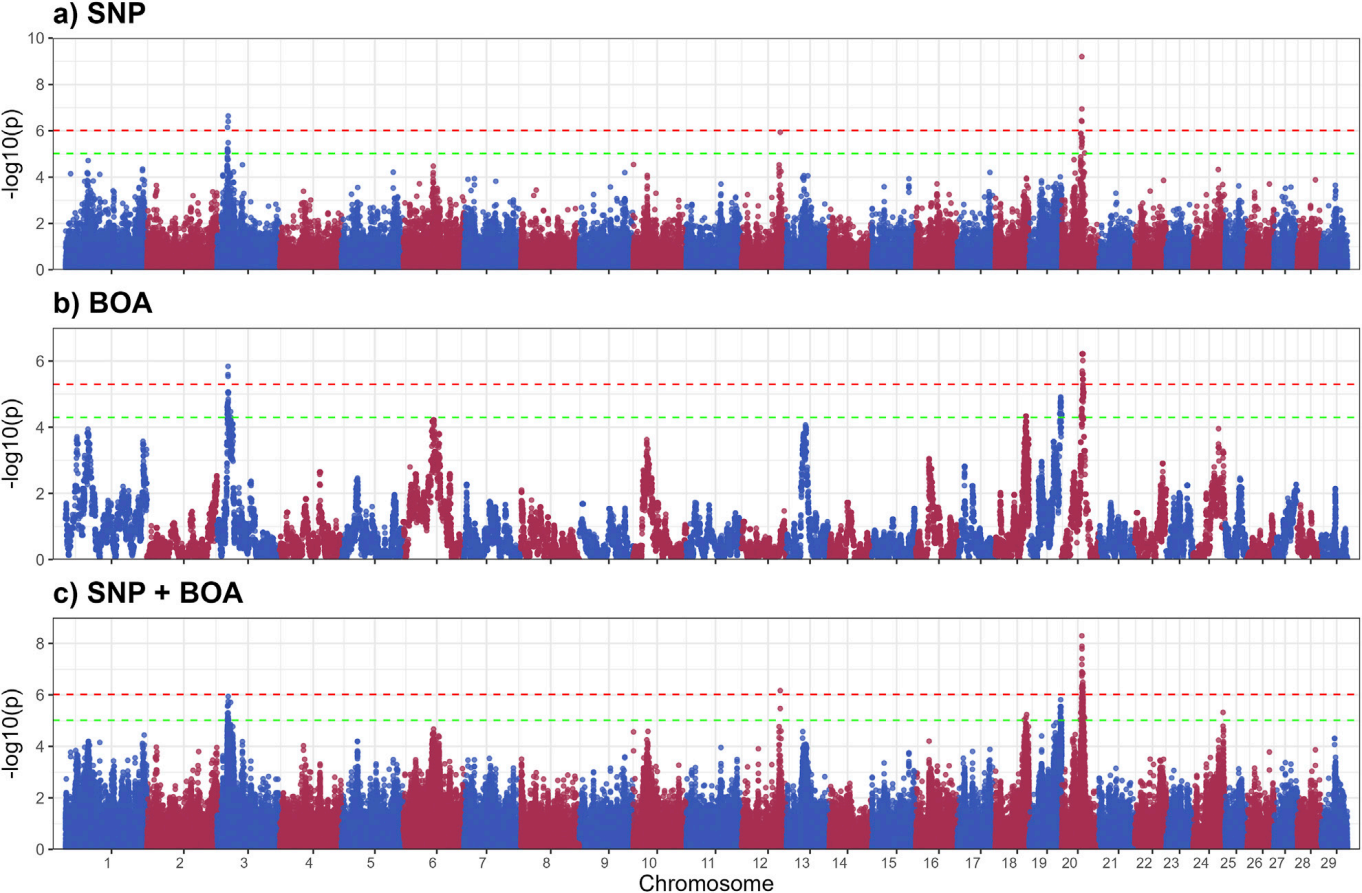
Manhattan plots from GWAS for short hair length using **(a)** SNP-based, **(b)** BOA-based, and **(c)** SNP + BOA combined analyses. The -log10(p-values) are plotted across chromosomes, with green and red dashed lines representing the suggestive threshold (1/number of SNPs) and genome-wide significance threshold (Bonferroni correction, α = 0.1), respectively.

Figure 2 displays the GWAS Manhattan plot for the SHL trait, with associated QTL intervals and significance levels provided in Table 2. For detailed information of SNP and BOA effects see Supplementary Table S2. Seven QTL were identified for SHL. Three QTL, located on BTA 3 (19.8–22.4 Mb), BTA 12, and BTA 20 surpassed the Bonferroni threshold and were classified as significant. The remaining four QTL located on BTA 3 (26.1 Mb region), BTA 18, BTA 19 and BTA 24 reached suggestive levels of significance.

The QTL on BTA20 was by far the most significant peak across both hair length traits. The alleles resulting in shorter hairs for both LHL and SHL were predominantly of Brahman origin, indicating a strong genetic contribution from this breed. However, the same allele was also observed in some Angus haplotypes, suggesting shared genetic variation between the breeds in this region of the genome. This QTL on BTA20, encompasses the prolactin receptor (*PRLR*) gene, which is strongly associated with hair length traits critical for thermoregulation (Sarlo Davila et al., 2019; Sosa et al., 2022). Shorter hair minimizes insulation and enhances heat dissipation, making this region a valuable target for breeding programs in hot climates (Olson et al., 2003; Dikmen et al., 2014; Sarlo Davila et al., 2019; Evangelista Façanha et al., 2020; Hansen, 2020). *PRLR* plays a role in the slick-hair phenotype observed in Criollo cattle, known to enhance thermotolerance (Dikmen et al., 2008; Huson et al., 2014; Porto-Neto et al., 2018; Sosa et al., 2021). The SLICK mutation in *PRLR* has been successfully introgressed into Holstein cattle, with the goal to improve temperature regulation in Holstein cattle in Florida, Unites States (Dikmen et al., 2008; Zayas et al., 2024a). Additionally, the *PRLR* gene has recently been successfully gene edited into Angus calves in an experimental setting, with the goal of improving thermotolerance in Angus cattle (Cuellar et al., 2024). Although the mutation identified by our study differs from the classic SLICK mutations, it likely performs a similar function in reducing hair length. This mutation was previously reported in a subset of this population, but its ancestral origin remained unclear (Sarlo Davila et al., 2020). Our model incorporating both BOA and SNP information found that a significant allele substitution effect −1.02 ± 0.13 whilst the BOA effect was 0.00 ± 0.11. Indicating that this effect for BOA was due to the higher prevalence of the beneficial allele rather than a BOA effect.

A unique QTL of interest for SHL is the QTL on BTA3 (19.7–22.3 Mb), overlapping *ECM1*, a biologically relevant gene encoding an extracellular matrix glycoprotein (Sercu et al., 2008). Mutations in *ECM1* are linked to alopecia, attributed to compromised skin basement membrane integrity and impaired hair follicle health (Vahidnezhad et al., 1993).

A suggestive SHL QTL was identified on BTA19 (59.87–63.43 Mb), where the BOA model revealed a significant Angus substitution effect (0.22 ± 0.05, rs42969983). Supplementary Figure S1 shows a more detailed Manhattan plot of this region and boxplots showing the effect of BOA. This region contains several functionally relevant genes related to hair development and cycle, including *ABCA5*, *PRKCA*, and *WIPI1*. *ABCA5* and its paralogs (*ABCA6*, *9*, *10*) are highly expressed in skin and hair follicles, with mutations linked to hypertrichosis and elongated follicles (DeStefano et al., 2014). Mutations in *ABCA5* are known to cause excessive hair overgrowth, with affected individuals exhibiting significantly longer and thicker hair follicles (DeStefano et al., 2014). *PRKCA* encodes PKC-α, a kinase upregulated during hair growth and expressed in the outer root sheath of anagen follicles, its overexpression in mouse models has been associated with accelerated keratinocyte proliferation and enhanced hair follicle growth (Li et al., 2003). *WIPI1*, an autophagy regulator, is critical for hair follicle cell survival and timing of hair cycle transitions (Parodi et al., 2018; Sun et al., 2024).

The QTL on BTA19 for LHL demonstrates the unique value of BOA in capturing genetic signals that are otherwise missed by traditional SNP-based analyses. This QTL exhibited significant Angus substitution effects 0.56 ± 0.12 for LHL (rs41922675), with Angus showing considerably longer hair relative to Brahman. Supplementary Figure S1 shows a more detailed Manhattan plot of this region and boxplots showing the effect of BOA. These findings align with well-documented breed differences in hair length (Sarlo Davila et al., 2019, 2020). Several genes in this region are involved in hair development and thermoregulation. These genes include *ACE* and *MAP3K3* which are involved in blood flow regulation and signaling pathways in response to stress. *ACE* plays a critical role in the renin-angiotensin system (RAS), which regulates blood flow, vascular tone, and inflammatory responses (Pontremoli et al., 1996; Karnik et al., 2009). Its association with hair follicle biology is underscored by its involvement in androgenetic alopecia (Karnik et al., 2009; Heymann, 2022). During stress, *ACE* redirects blood flow to vital organs by changing vaso- dilation and constriction, reducing blood supply to hair follicles thus resulting in shorter hair length (Yang et al., 1996; Saavedra and Benicky, 2007). Similarly, *MAP3K3* acts as an upstream regulator of stress-activated protein kinases, such as the c-Jun N-terminal kinase (*JNK*) and p38 *MAPK* pathways. These pathways influence hair follicle biology by regulating keratinocyte function and inflammatory responses, which are crucial for hair growth and cycling (Chuang et al., 2000; Botchkareva et al., 2006; Quan et al., 2019). Dysregulated *MAP3K3* activity could lead to premature hair follicle regression, impacting hair growth and length. This is further supported by the fact that the KEGG enrichment analysis on suggestive and significant genes for LHL, found enrichment for the MAPK signaling pathway. Another gene in this region, *CCDC47* has been linked to wooly hair in humans, highlighting its potential role in hair structure and morphology (Morimoto et al., 2018). While the precise mechanism remains unclear, its involvement in calcium ion homeostasis may influence keratinocyte function and hair follicle health (Morimoto et al., 2018). Given that Brahman origin is beneficial in this region and there is a presence of multiple biologically relevant genes, we speculate that the observed effect arises from several advantageous variants distributed across these genes rather than a single causative mutation This complexity explains why the BOA analysis, which captures the combined effects of ancestral haplotypes could identified this region. Whereas the traditional SNP analysis which relies on individual marker effects or markers in LD (often disrupted in crossbred populations) failed to detect the effect.

### 3.3 GWAS sweat gland area

**FIGURE 3:**
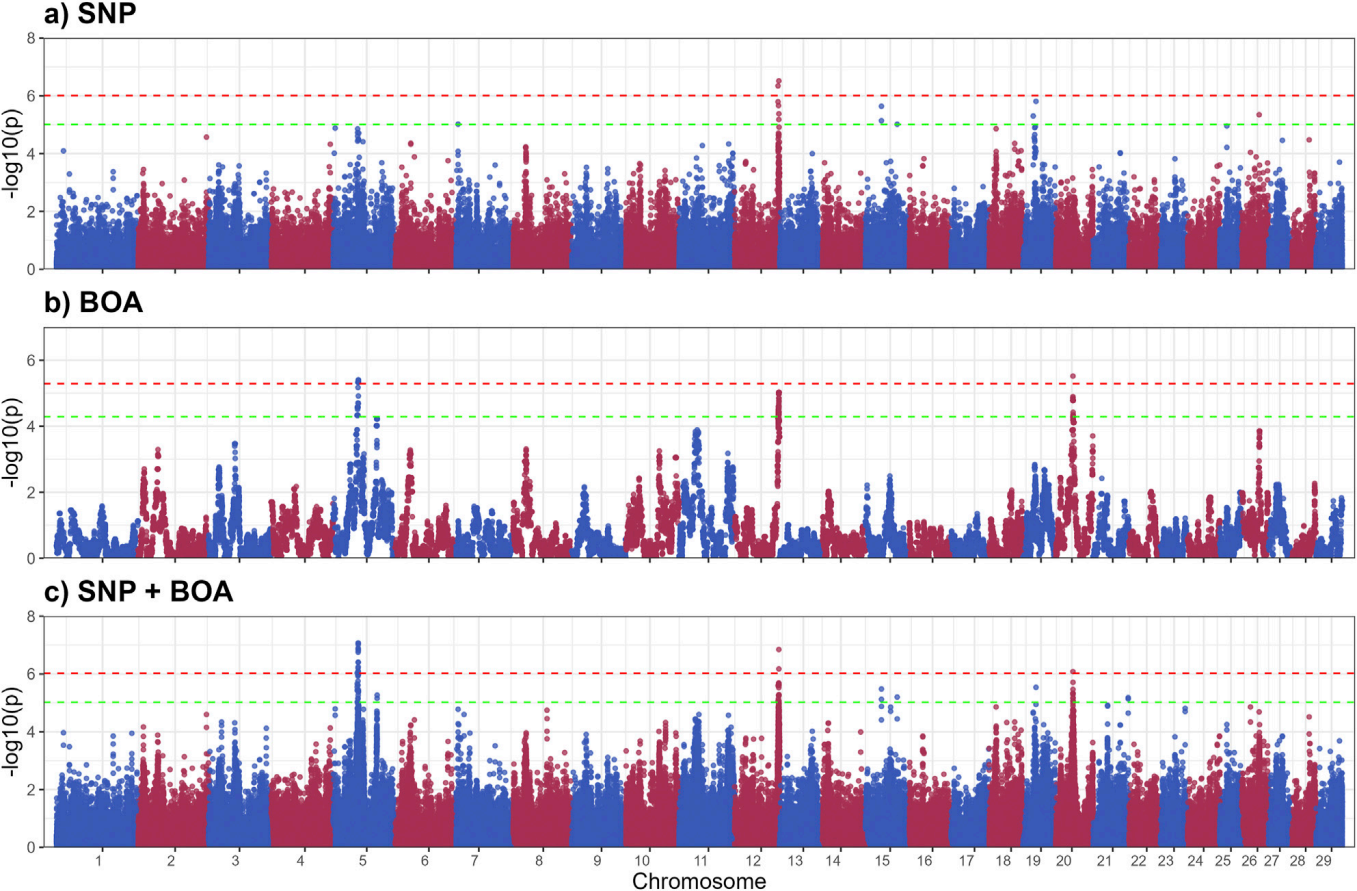
Manhattan plots from GWAS for sweat gland area using **(a)** SNP-based, **(b)** BOA-based, and **(c)** SNP + BOA combined analyses. The -log10(p-values) are plotted across chromosomes, with green and red dashed lines representing the suggestive threshold (1/number of SNPs) and genome-wide significance threshold (Bonferroni correction, α = 0.1), respectively.

The GWAS results for SGA are presented in Figure 3, with putative QTLs summarized in Table 2 and significant markers detailed in Supplementary Table S3. A total of ten QTLs were identified, with three significant QTLs on BTA 5,13 and 20 and 7 QTLs across BTA 5,7,15,19,2 and 26. These QTLs were predominantly associated with BOA effects, with some regions also showing combined SNP and BOA contributions. Across all identified QTLs, Angus BOA inheritance was consistently associated with a decrease in SGA, as shown in Supplementary Table S3. This pattern underscores the ancestral influence of Angus genetics on sweat gland morphology, suggesting a potential evolutionary adaptation or breed-specific trait contributing to this characteristic.

Modeling the SNP and BOA effects simultaneously, demonstrated superior resolution in identifying QTLs compared to the SNP-only and the BOA-only models. This integrated approach revealed combined effects in genomic regions that were less distinct when analyzed using either SNP or BOA effects independently. By accounting for both additive and breed-specific contributions, we were able to gain deeper insights into the genetic architecture of complex traits such as SGA.

The significant QTL on BTA 5 includes several genes with known roles in skin and sweat gland development, notably *WIF1*, *GRIP1*, *MSRB3*, and *LEMD3*. *WIF1* encodes a protein that antagonizes Wnt signaling by binding to Wnt proteins (Hsieh et al., 1999). As Wnt signaling is essential for the formation and differentiation of ectodermal appendages, including sweat glands (Lu et al., 2012), dysregulation of *WIF1* may impact sweat gland morphogenesis, size, and function (Coates et al., 2019). *GRIP1* encodes a scaffolding protein that organizes transmembrane receptors at the cell membrane. It plays a crucial role in epidermal basement membrane attachment. Mutations in *GRIP1* cause Fraser syndrome, characterized by skin syndactyly and dermo-epidermal junction defects; in mouse models, loss of *GRIP1* results in epidermal blistering due to impaired dermal-epidermal junction integrity (Vogel et al., 2012).

*MSRB3* encodes a methionine sulfoxide reductase involved in repairing oxidatively damaged proteins, thereby protecting skin cells from oxidative stress. It is expressed in vascular endothelial cells of the skin and contributes to dermal integrity and aging processes (Taungjaruwinai et al., 2009). Lastly, *LEMD3* encodes the inner nuclear membrane protein MAN1, which antagonizes TGF-β/BMP signaling through Smad binding. This regulation is critical for connective tissue and bone homeostasis. Loss-of-function mutations in *LEMD3* cause Buschke-Ollendorff syndrome, characterized by connective tissue nevi and osteopoikilosis, indicating a requirement for *LEMD3* in normal dermal connective tissue development (Hellemans et al., 2004). BOA effects showed that Angus ancestry significantly reduced SGA (−23.28 ± 4.37,rs210687570), consistent with previous findings linking Angus genetics to smaller sweat glands and reduced heat dissipation efficiency (Mateescu et al., 2023; Hernandez et al., 2024). This suggests that targeting Brahman-derived alleles could be advantageous for improving thermotolerance in cattle.

### 3.4 GWAS thermal stress slope

TSS reflects an animal’s ability to maintain stable core body temperature under heat stress, serving as a critical metric for thermotolerance. Animals with lower TSS values demonstrate better thermoregulation, experiencing smaller fluctuations in body temperature under heat stress. In contrast, higher TSS values indicate reduced thermoregulatory capacity due to less efficient responses to heat load. This trait, which accounts for daily body temperature variations and cumulative heat stress exposure, is pivotal for understanding the genetic basis of thermoregulation and guiding breeding strategies aimed at enhancing heat resilience.

**FIGURE 4:**
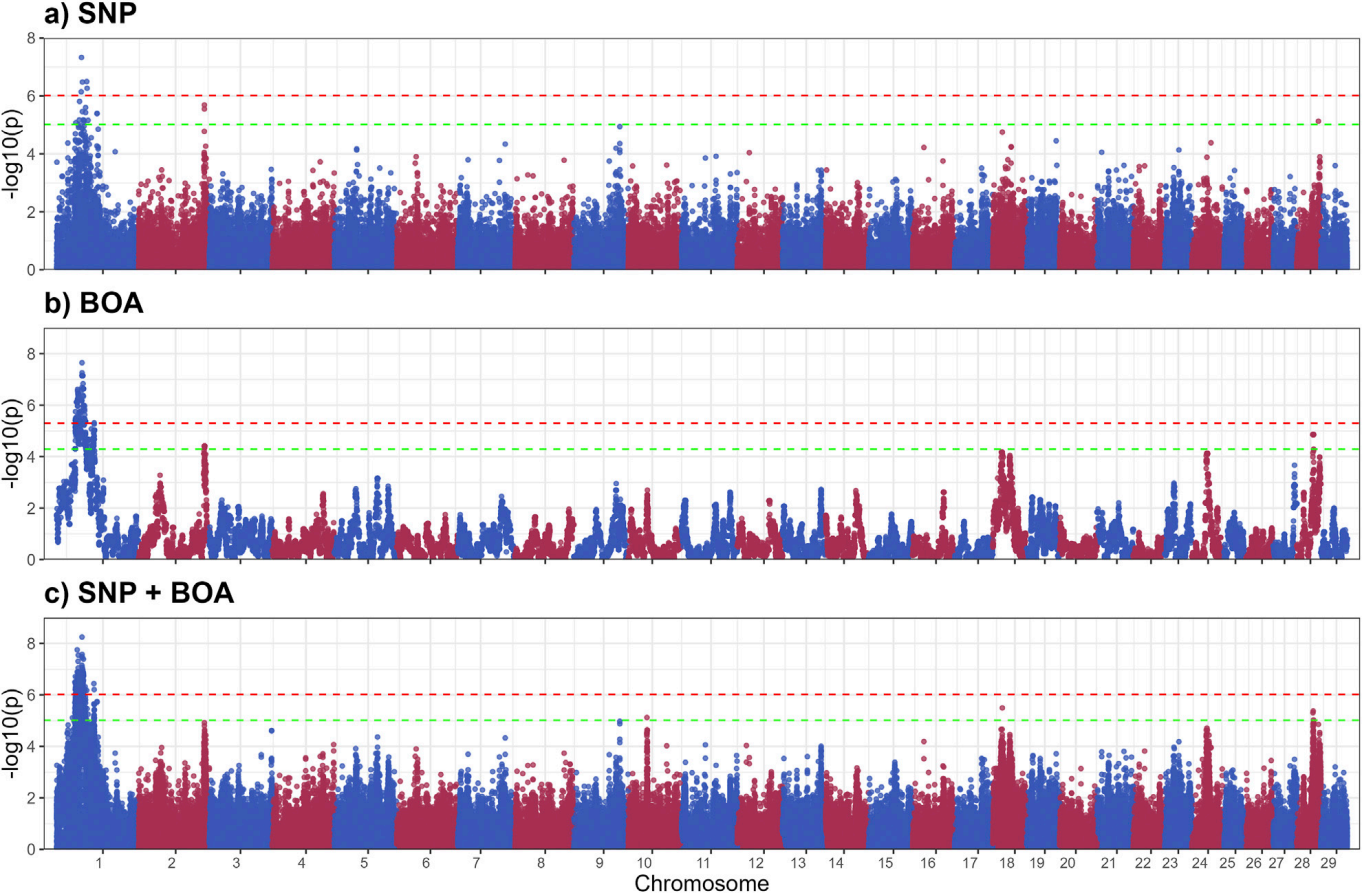
Manhattan plots from GWAS for thermal stress slope (TSS) using **(a)** SNP-based, **(b)** BOA-based, and **(c)** SNP + BOA combined analyses. The -log10(p-values) are plotted across chromosomes, with green and red dashed lines representing the suggestive threshold (1/number of SNPs) and genome-wide significance threshold (Bonferroni correction, α = 0.1), respectively.

The GWAS results for TSS, presented in Figure 4, identified nine QTLs, of which 4 significant QTLs were found on BTA1. Five suggestive QTLs were found on BTA 2,19,18 and 28. Supplementary Table S4 provides details of suggestive and significant markers.

The QTL on BTA1(44.0–58.5 Mb) spans a large genomic region (Table 2) containing multiple genes involved in stress response and adaptation. Among these, *FILIP1L* and *ALCAM* are particularly notable for their interactions with heat shock proteins and pathways related to immune and heat stress responses. The *FILIP1L* gene encode for a Heat shock factor (Hsf1) interacting protein, which reduces Hsf1-mediated transcription Hsf1 by promoting Hsf1 ubiquitination and degradation (Hu and Mivechi, 2011). Heats shock proteins are vital to heat stress response, these results indicate that Brahman BOA may have variants that better regulate the expression of heat shock proteins. The Activated Leukocyte Cell Adhesion Molecule (*ALCAM)* gene plays a role in immune cell adhesion and migration, which is critical for immune function, particularly under stress conditions like heat exposure (Zimmerman et al., 2006; te Riet et al., 2007; Chan et al., 2015). Heat stress can upregulate integrin adhesion molecules, which interact closely with *ALCAM* to enhance leukocyte adhesion and migration (Chan et al., 2015; Tukaj, 2020). Heat shock protein 70 (HSP70), a molecular chaperone, plays a critical role in stabilizing cell adhesion proteins during thermal stress, enhancing leukocyte function and adhesion (te Riet et al., 2007; Chan et al., 2015; McRobb et al., 2019). HSP70 stabilizes cell adhesion proteins, potentially enhancing leukocyte functionality and adhesion under heat stress conditions (Chan et al., 2015; Rigg et al., 2016). The interaction between *ALCAM*, integrins, and HSP70 supports immune function and helps mitigate thermal damage, contributing to immune resilience during heat stress. Other genes related to immune and stress response include the *NFKBIZ* and *DCBLD2* genes. *NFKBIZ* functions as a regulator in the NF-κB signaling pathway, which is crucial for immune and stress responses (Wong et al., 1997; Malhotra et al., 2002). Alterations in NF-κB signaling have been linked to heat stress responses in livestock. *DCBLD2*, a transmembrane protein with roles in cellular proliferation and stress responses, has been implicated in oxidative stress pathways a consequence of heat stress (Chen et al., 2021; Wang et al., 2024). The Angus substitution effect of this QTL was estimated at 0.27 ± 0.05 (rs3423104800), indicating that Angus inheritance has a detrimental impact on thermotolerance. A more detailed Manhattan plot of the QTL alongside other QTLs on BTA 1 can be found in Supplementary Figure S2. The observed effect of Angus alleles in this region, associated with an unfavorable increase in TSS, suggests that Brahman BOA may offer genetic advantages for heat resilience. Integrating the SNP and BOA effects, demonstrated superior resolution in disentangling the overlapping contributions of these factors. This integrated approach not only improves the precision of QTL mapping but also offers deeper insights into how breed-specific and additive genetic factors interact to influence complex traits like thermoregulatory capacity under heat stress.

### 3.5 Functional enrichment analysis

To further explore the biological relevance of the genomic regions associated with thermotolerance traits, gene ontology (GO) and KEGG pathway enrichment analyses were conducted for the set of candidate genes identified within significant and suggestive QTLs for each trait.

For LHL, enriched GO biological processes included *keratinocyte differentiation* (GO:0030216, FDR = 0.001), *skin development* (GO:0043588, FDR = 0.001), and *keratinization* (GO:0031424, FDR = 0.003). These terms reflect the biological underpinnings of hair growth and structure. Additionally, KEGG pathway analysis revealed enrichment for the *MAPK signaling pathway* (bta04010, FDR = 0.047) and *estrogen signaling pathway* (bta04915, FDR = 0.045), both implicated in skin and hair follicle regulation (Oh and Smart, 1996; Ohnemus et al., 2005; Akilli Öztürk et al., 2015; Lu et al., 2021). For SHL, GO analysis highlighted terms related to *epidermis morphogenesis* (GO:0048730), *cell-cell adhesion* (GO:0098742), and *adherens junction organization* (GO:0034332), alongside molecular functions such as *DNA-binding transcription repressor activity* and *calcium-activated chloride channel activity*. KEGG results showed significant enrichment for *adherens junctions* (bta04520), consistent with the involvement of adhesion molecules and cytoskeletal elements in follicular structure and integrity (Young et al., 2003; Wu et al., 2014).

The SGA trait exhibited highly significant enrichment (FDR < 0.001) for multiple GO terms associated with *epidermal and epithelial development*, including *epidermis development* (GO:0008544), *keratinocyte differentiation* (GO:0030216), and *intermediate filament organization* (GO:0045109). These findings align with the morphological nature of sweat gland area and its dependence on dermal structure.

For TSS, GO terms associated with *skin development* (GO:0043588, FDR = 0.013), *keratinization* (GO:0031424, FDR = 0.013), and *innate immune responses* (GO:0002220, FDR = 0.038) were enriched. These results underscore the dual role of skin in thermoregulation and immune response under heat stress. KEGG pathways significantly enriched for TSS included the *MAPK signaling pathway* (bta04010, FDR = 0.047) and *estrogen signaling pathway* (bta04915, FDR = 0.045), both relevant to heat shock response and cellular adaptation (Stice and Knowlton, 2008; Kumar et al., 2020; Hu et al., 2023).

Overall, enrichment results corroborate the trait-specific biological processes and pathways highlighted in our GWAS findings. The consistent enrichment of skin- and keratin-related processes across LHL, SHL, and SGA, and immune/stress response pathways for TSS, reinforce the validity of the detected QTLs and support the functional relevance of the implicated genes in thermotolerance phenotypes.

## 4 Broader implications and limitations

This study builds upon previous research utilizing BOA, which demonstrated the value of ancestral information for genomic evaluations and QTL identification (Guillenea et al., 2023; Warburton et al., 2023; Hernandez et al., 2024; Zayas et al., 2024b). The integrated modeling strategy provides a more comprehensive understanding of genetic contributions, as demonstrated by the identification of suggestive and significant QTLs for thermotolerance traits. This approach differs from studies that have modeled SNPs in a breed-specific manner without directly incorporating BOA effects (Eiríksson et al., 2021; Guillenea et al., 2023; Warburton et al., 2023). By leveraging BOA, we identified significant QTLs for thermotolerance traits that were undetectable using SNP-only approaches. These findings highlight the critical role of breed-specific genetic contributions in shaping thermotolerance traits. For instance, Brahman-derived alleles were associated with improved thermoregulation, while Angus-derived alleles were linked to traits such as longer hair and reduced sweat gland area. This detailed understanding of ancestral effects provides actionable insights for breeding programs. Specifically, targeted selection for Brahman-derived alleles represents a promising strategy to enhance heat resilience in cattle in tropical climates.

Future studies are needed to investigate the mechanisms behind the BOA effects observed for these traits. We hypothesize that BOA may provide a more accurate model for QTLs in crossbred cattle compared to SNP-based approaches alone. Another possibility is that BOA has the potential to capture non-SNP factors, such as regulatory or epigenetic mechanisms, that influence gene expression and phenotypes. Because BOA assigns variants to their ancestral origin, it provides a framework for disentangling breed-specific regulatory effects in crossbred populations. Integrating BOA analyses with functional genomic tools such as transcriptomics, eQTL mapping, RNA-Seq, Chip-Seq or ATAC-Seq could enable the identification of regulatory elements or expression patterns that are unique to a particular ancestral background. This breed-aware regulatory mapping could uncover ancestry-specific transcription factor binding sites, epigenetic marks, or chromatin accessibility profiles that underlie observed phenotypic differences. Such analyses may offer a deeper understanding of the genetic and regulatory networks driving complex traits, ultimately improving biological interpretation and improving the precision of breeding strategies.

Despite its potential, the application of BOA has certain limitations. The resolution of BOA assignments is constrained by the density of SNP arrays, which may miss finer-scale ancestral contributions. Future studies utilizing whole-genome sequencing or high-density genotyping arrays could enhance the precision and accuracy of BOA analyses, providing a more detailed understanding of ancestral genetic influences. Furthermore, the computational complexity of integrating BOA with SNP-based approaches underscores the need for efficient analytical pipelines to support wider adoption.

Incorporating BOA into genomic studies represents a powerful tool for advancing our understanding of the genetic architecture of complex traits using crossbred and composite populations. By leveraging breed-specific ancestry information, this approach offers valuable insights into the complex interactions between ancestry and phenotype. It paves the way for a more nuanced understanding of the genetic dynamics within these populations, ultimately supporting more effective breeding and selection strategies.

## 5 Conclusion

This study demonstrates the power of BOA for dissecting the genetic architecture of thermotolerance using crossbred cattle. By integrating BOA with SNP effects, we uncovered novel QTL and ancestral contributions that are critical for improving thermoregulation in cattle. For LHL and SHL, a major QTL on BTA20 overlapping *PRLR* showed strong associations with shorter hair, with beneficial alleles predominantly of Brahman origin. For SGA, Angus BOA consistently decreased gland size, with a significant BTA5 QTL containing genes such as *WIF1*, *GRIP1*, and *LEMD3*, all involved in skin structure and development. The most significant TSS QTL on BTA1 harbored *FILIP1L* and *ALCAM*, which regulate immune response and interact with heat shock proteins during thermal stress. Functional enrichment revealed MAPK and estrogen signaling pathways were common to both LHL and TSS, underscoring shared regulatory mechanisms. These results highlight BOA’s ability to uncover breed-specific genetic effects missed by SNP-only models. These findings provide valuable insights for breeding programs aiming to enhance heat resilience of beef cattle in tropical and subtropical climates. Future research should aim to validate these findings and explore functional mechanisms underlying the observed QTL, maximizing their potential for use in genetic improvement strategies.

## Data Availability

The genotypic data is available in the European Variation Archive repository, PRJEB75981. Scripts used for analysis are available on Github https://github.com/gzayasPR/BOA_GWAS and https://github.com/gzayasPR/BOAxSNP-GWAS.git.
