# Supplementary material for: Genetic architecture of thermotolerance traits in beef cattle: a novel integration of SNP and breed-of-origin effects
Source: Front Genet. 2025 Apr 30;16:1576966. doi: 10.3389/fgene.2025.1576966 (PMC12075150; doi:10.3389/fgene.2025.1576966)
Supplement: Supplementary file 2 [file DataSheet1.docx]

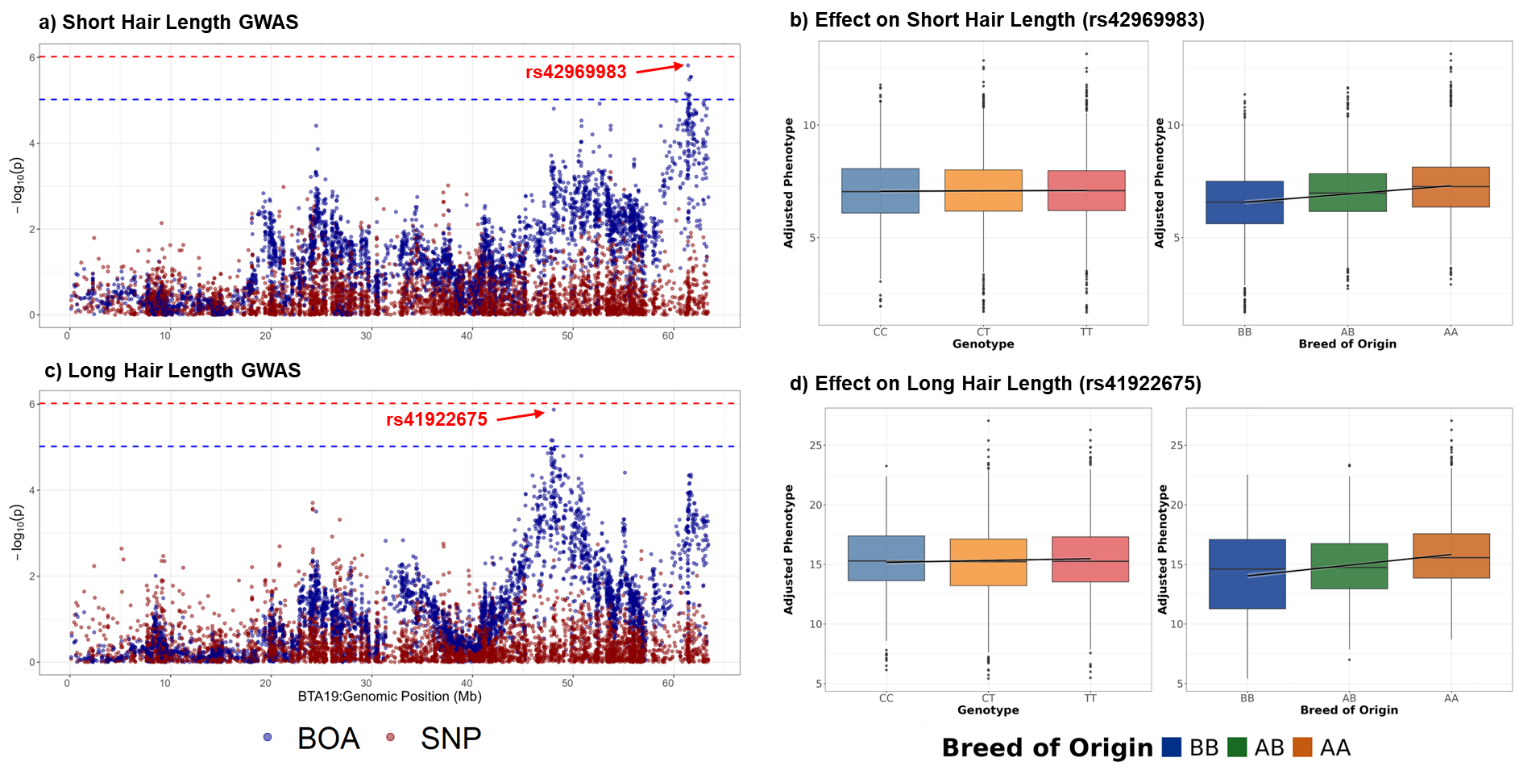


Supplementary Figure1). Genome-wide association results and effects of genotype and Breed of Origin (BOA) on hair length trait from model 3. (a) Manhattan plot for short hair length GWAS showing -log10(p-values) for SNP (red) and BOA (blue) across chromosome 19, with dashed horizontal lines indicating the suggestive (blue) and genome-wide significance (red) thresholds. (b) Boxplots showing the effect of SNP genotype (CC, CT, TT) and BOA (BB, AB, AA) on adjusted short hair length phenotype. (c) Manhattan plot for long hair length (LHL) GWAS, highlighting significant SNP and BOA signals on chromosome 19. Thresholds for significance are the same as in (a). (d) Boxplots depicting the effect of SNP genotype and BOA on adjusted long hair length phenotype, illustrating the contribution of breed-specific ancestry and genotype to phenotypic variation.

Breed of Origin categories: BB (Brahman-Brahman), AB (Angus-Brahman), and AA (Angus-Angus).


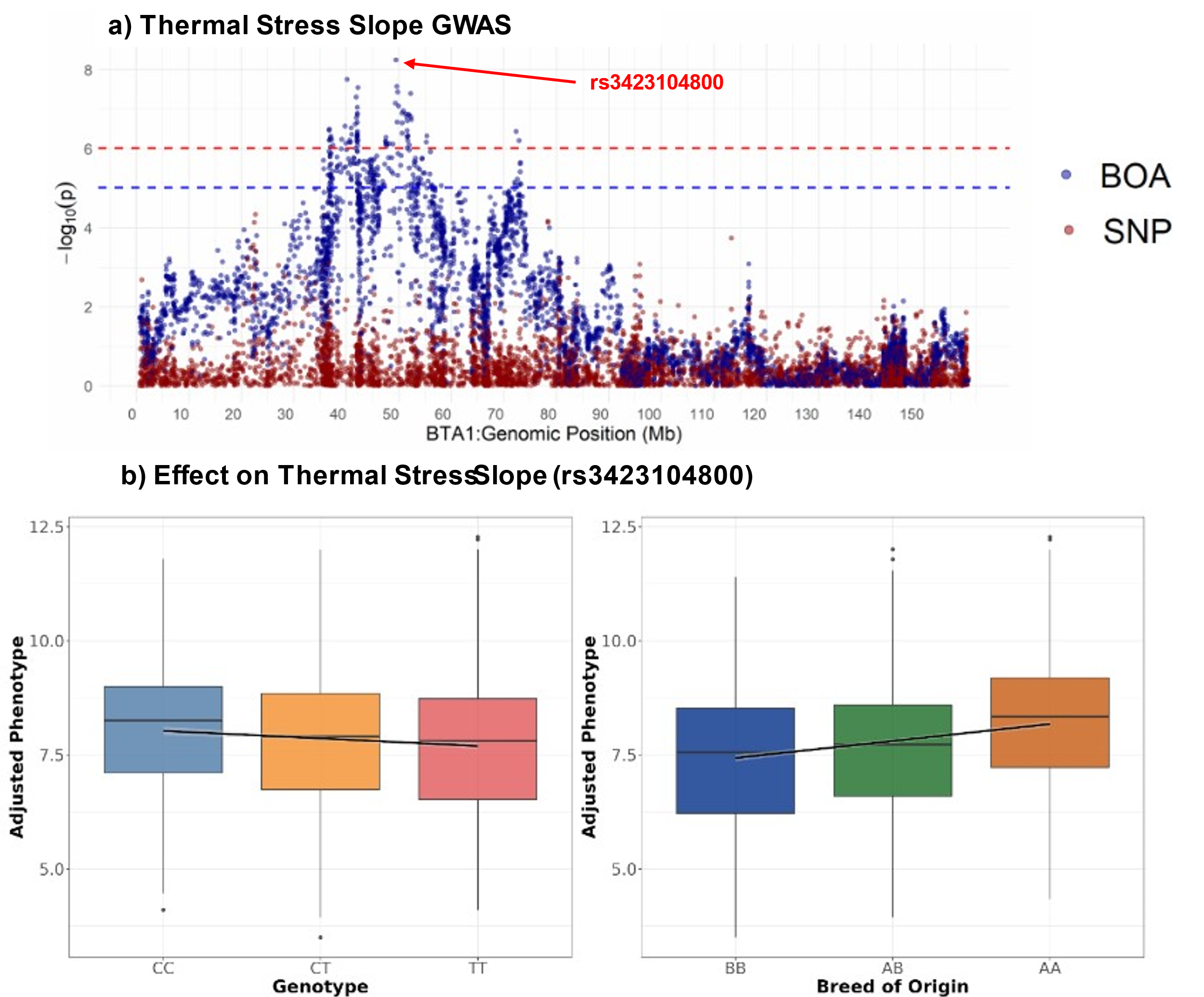


Supplementary Figure 2) Genome-wide association results and effects of genotype and Breed of Origin (BOA) on thermal stress slope (TSS). (a) Manhattan plot for TSS GWAS displaying -log10(p-values) for SNP (red) and BOA (blue) on chromosome 1. Horizontal dashed lines indicate the suggestive (blue) and genome-wide significance (red) thresholds. A prominent QTL is observed at approximately 50 Mb, identified using BOA effects. (b) Boxplots showing the effect of SNP genotype (CC, CT, TT) and BOA (BB, AB, AA) on the adjusted thermal stress slope phenotype. The boxplots highlight the contribution of both genotype and breed-specific ancestry to phenotypic variation in TSS, with Angus ancestry (AA) associated with higher TSS values.

Breed of Origin categories: BB (Brahman-Brahman), AB (Angus-Brahman), and AA (Angus-Angus).
